# “Bot or Not”: Turing Problem in Otolaryngology

**DOI:** 10.7759/cureus.48170

**Published:** 2023-11-02

**Authors:** Aynur Aliyeva

**Affiliations:** 1 Otolaryngology - Head and Neck Surgery, Cincinnati Children's Hospital Medical Center, Ohio, USA

**Keywords:** bot or not, artificial intelligence, turing problem, otolaryngology, chatgpt

## Abstract

The aim of this article is to shed light on the evolving landscape of artificial intelligence (AI) integration in otolaryngology and its implications, particularly focusing on the ethical considerations surrounding AI applications, and to highlight the potential benefits of ChatGPT in patient management and scientific research within otolaryngology while emphasizing the necessity for ethical guidelines and validation processes. Ultimately, the article seeks to encourage a responsible and informed approach to AI adoption in otolaryngology, promoting collaboration between AI and healthcare professionals for the betterment of science and human well-being.

## Editorial

This article aims to shed light on an intriguing and rapidly advancing domain, entwining artificial intelligence (AI) and otolaryngology, with the spotlight on the Turing Test. Alan Turing, in 1950, brought forth a benchmark to ascertain a machine's ability to exhibit intelligent behavior equivalent to, or indistinguishable from, that of a human. The question that remains, "Bot or Not," particularly strikes a chord in the otolaryngology field, where AI integration, exemplified by ChatGPT, is increasingly palpable [1,2].

Neoethics of ChatGPT in science

As the utilization of AI becomes pervasive in scientific research and healthcare, the neuroethics surrounding its application demands scrupulous attention. ChatGPT, a prodigious language model, has showcased significant potential in aiding research, patient management, and generating scientific content. However, a prudent exploration of ethical boundaries is imperative to ensure responsible usage, data privacy, and avoidance of misinformation.

ChatGPT's integration into science beckons several ethical considerations. The integrity of scientific content generated by AI models necessitates rigorous validation to prevent the dissemination of inaccuracies. Furthermore, equitable access to AI technologies, transparency in their functionality, and accountability in case of erroneous recommendations are pivotal aspects of the neuroethical framework [3].

Role of ChatGPT in otolaryngology patient management

In otolaryngology, ChatGPT is carving a niche in augmenting patient management. Its ability to sift through copious amounts of medical data and literature aids in formulating tailored treatment plans, thereby enhancing personalized medicine. For patients presenting with ailments ranging from hearing impairment to head and neck cancers, ChatGPT offers insightful, evidence-based recommendations, serving as a valuable adjunct to the clinician's expertise.

Moreover, ChatGPT is instrumental in patient education and engagement. Offering comprehensible explanations and addressing queries bridge the communication gap and foster an enriched patient-doctor relationship. The convenience and accessibility of AI-driven solutions, such as ChatGPT, amplify healthcare outreach, which is particularly beneficial in resource-limited settings.

ChatGPT in otolaryngology scientific paper production

ChatGPT's integration into otolaryngology scientific paper production exemplifies AI's growing role in specialty research. With its advanced language capabilities, ChatGPT assists in drafting manuscripts, literature reviews, and proposals, catering to the specific needs of otolaryngological studies. Emerging trends include using ChatGPT for rapid literature reviews, meta-analyses, and interpreting complex datasets, streamlining the extraction of meaningful insights. The technology also enhances multidisciplinary collaboration in otolaryngology by fostering seamless communication among professionals. Despite these advancements, challenges such as ensuring content accuracy necessitate meticulous human review. Addressing these challenges is vital for maintaining scientific integrity and leveraging ChatGPT's potential in advancing otolaryngology research [4].

Collaboration for human well-being

In conclusion, the confluence of AI, epitomized by ChatGPT and otolaryngology, is laden with opportunities and challenges. The Turing problem remains a testament to the evolving capabilities of AI as it becomes increasingly entwined with various facets of healthcare and scientific research.

The collaboration between ChatGPT and healthcare professionals holds immense promise for human well-being. By addressing ethical considerations, refining the integration of AI in patient management, and ensuring the reliability of AI-generated scientific content, we can embark on a journey toward enhanced healthcare delivery and scientific discovery.

The interplay of human expertise and artificial intelligence is the linchpin for advancing healthcare frontiers. By embracing the strengths of ChatGPT and mitigating its limitations through ethical practices and continuous learning, the field of otolaryngology, and, by extension, the broader spectrum of healthcare, stands on the precipice of a transformative era [5].

The visionary words of Alan Turing resonate now more than ever as we navigate the intricate tapestry of "Bot or Not," exploring the realms of possibilities that the collaboration between ChatGPT and humans can unravel for the betterment of science and human well-being.
